# Targeted liquid chromatography tandem mass spectrometry to quantitate wheat gluten using well-defined reference proteins

**DOI:** 10.1371/journal.pone.0192804

**Published:** 2018-02-09

**Authors:** Kathrin Schalk, Peter Koehler, Katharina Anne Scherf

**Affiliations:** Leibniz-Institute for Food Systems Biology at the Technical University of Munich, Freising, Germany; Institute of Genetics and Developmental Biology Chinese Academy of Sciences, CHINA

## Abstract

Celiac disease (CD) is an inflammatory disorder of the upper small intestine caused by the ingestion of storage proteins (prolamins and glutelins) from wheat, barley, rye, and, in rare cases, oats. CD patients need to follow a gluten-free diet by consuming gluten-free products with gluten contents of less than 20 mg/kg. Currently, the recommended method for the quantitative determination of gluten is an enzyme-linked immunosorbent assay (ELISA) based on the R5 monoclonal antibody. Because the R5 ELISA mostly detects the prolamin fraction of gluten, a new independent method is required to detect prolamins as well as glutelins. This paper presents the development of a method to quantitate 16 wheat marker peptides derived from all wheat gluten protein types by liquid chromatography tandem mass spectrometry (LC-MS/MS) in the multiple reaction monitoring mode. The quantitation of each marker peptide in the chymotryptic digest of a defined amount of the respective reference wheat protein type resulted in peptide-specific yields. This enabled the conversion of peptide into protein type concentrations. Gluten contents were expressed as sum of all determined protein type concentrations. This new method was applied to quantitate gluten in wheat starches and compared to R5 ELISA and gel-permeation high-performance liquid chromatography with fluorescence detection (GP-HPLC-FLD), which resulted in a strong correlation between LC-MS/MS and the other two methods.

## Introduction

Celiac disease (CD) is an inflammatory disorder of the upper small intestine in genetically predisposed individuals. It is triggered by the ingestion of storage proteins from wheat (gliadins, glutenins), rye (secalins), barley (hordeins), and possibly oats (avenins) that are called gluten in the field of CD. Typically, CD patients develop a flat intestinal mucosa (villous atrophy) resulting in malabsorption of nutrients together with extra- and intraintestinal symptoms [1]. Consequently, the only effective therapy for CD patients is to follow a strict gluten-free diet to prevent long-term consequences such as anemia, edema, osteoporosis, infertility, T-cell lymphoma, and other malignancies. The daily intake of gluten may not exceed 20 mg [2] and, therefore, CD patients need to consume gluten-free products which contain less than 20 mg gluten/kg according to Codex Standard 118–1979 [3]. To ensure the safety of gluten-free products, it is essential that appropriate analytical methods with high specificity and sensitivity are available. Enzyme-linked immunosorbent assays (ELISAs) are most frequently used by e.g. food manufacturers or control authorities to verify the gluten content in food products. Several ELISA kits for gluten detection are established on the market and the majority is based on the Skerritt (401.21) [4], R5 [5], G12 [6], and α20 [7] monoclonal antibodies. Currently, the ELISA based on the R5 monoclonal antibody is endorsed by legislation as Codex Alimentarius type 1 method [8]. Most of the antibodies are assumed to detect only prolamins, the gluten fraction soluble in aqueous alcohols. As a consequence, the gluten content is calculated by multiplying the prolamin content by a factor of 2, because the prolamin content of gluten is taken as 50% [3]. Several studies demonstrated that this calculation of the gluten content resulted in an over- or underestimation of gluten [9] which is mostly caused by different prolamin/glutelin ratios depending on the type of grain and the degree of food processing [10,11]. Due to this over- or underestimation of the gluten content by ELISA, new independent methods are urgently needed to verify the results determined by ELISA and to identify the source of gluten.

Currently, gluten analysis by mass spectrometry is the most promising non-immunochemical approach to ensure the safety of gluten-free products. Several approaches to the quantitation of gluten marker peptides by targeted liquid chromatography tandem mass spectrometry (LC-MS/MS) were published in recent years [12–16]. Sealey-Voyksner et al. (2010) developed an LC-MS/MS method to detect six CD-immunogenic wheat marker peptides in a range of 0.01 to 100 mg/kg in native and processed food samples. The method was calibrated by spiking a cocktail of six target peptides into proteolyzed corn flour at different concentrations [12]. Studies by Fiedler et al. (2014) demonstrated the development of a targeted LC-MS/MS approach based on two wheat marker peptides from α-gliadins to detect wheat contamination in oats. For this purpose, wheat flour was spiked into gluten-free oat flour to produce flour mixtures containing 10000 to 1 μg/g of wheat [13]. A further approach enabled proteomic profiling of 16 cereal grains and the quantitation of four wheat marker peptides down to 15 mg gluten/kg in wheat-contaminated soy flour [14]. Furthermore, nine CD-immunogenic peptides from α-gliadins were quantitated by van den Broeck et al. (2015) using LC-MS/MS. The calibration was performed by spiking a cocktail of nine marker peptides into a tryptic digest of a wheat gluten extract or of bovine serum albumin [15]. Although many studies reported the quantitation of gluten marker peptides [16], the calculation of gluten contents based on the obtained peptide concentrations was not attempted or achieved so far. All the illustrated LC-MS methods described an external calibration procedure by spiking peptides, gluten or gluten-containing flour into a gluten-free matrix. The quantitation was neither performed based on the addition of an internal peptide standard nor were defined gluten reference proteins used.

This paper demonstrates a novel strategy to define wheat gluten marker peptides as well as the development of a targeted LC-MS/MS method for the quantitative determination of 16 wheat marker peptides, which were specific for each wheat gluten protein type. The quantitation of marker peptides in well-characterized wheat reference proteins enabled the conversion of peptide into protein concentrations to quantitate gluten concentrations using an independent non-immunochemical method.

## Materials and methods

### Chemicals

The quality of all chemicals was of analytical grade or stated otherwise. Water for high-performance liquid chromatography (HPLC) was purified using an Arium 611VF water purification system (Sartorius, Goettingen, Germany). Disodium hydrogen phosphate dihydrate, ethanol, formic acid (FA; 98–100%), hydrochloric acid (32%), *n*-pentane, 1-propanol, potassium dihydrogen phosphate, sodium chloride, tris(hydroxymethyl)-aminomethane (TRIS), and urea were purchased from Merck (Darmstadt, Germany). α-Chymotrypsin (from bovine pancreas, TLCK-treated, ≥ 40 U/mg protein) and triflouroacetic acid (TFA; 99%) were obtained from Sigma-Aldrich (Steinheim, Germany. Acetonitrile (LC-MS-grade) was purchased from CLN (Freising, Germany). The wheat marker peptides (P1-16) and the isotopically labelled peptide LQLQPFPQPQLPYPQPQP*F* (*P11) with P*: L-[^13^C_5_][^15^N]-proline and F*: L-[^13^C_9_][^15^N]-phenylalanine (Table 1), were purchased from Genscript (Hongkong, PR China) with a purity of > 90%.

**Table 1:** Selected wheat marker peptides. Amino acid sequences of the 16 peptides (P1-16), their specificity for wheat gluten protein types, and the detected peptide scores in the flour.

Peptide	Amino acid sequence	Specificity (protein type)	Score^1^	NCBI Accession^2^
P1	QQQPLPPQQTFPQQPL	LMW-GS	41	ABD72601.1
P2	GQQPQQQQL	LMW-GS	33	AGK83348.1
P3	VQQQIPVVQPSIL	LMW-GS	30	ACF93464.1
P4	SIILQEQQQGF	LMW-GS	71	ACA63873.1
P5	LQPGQGQQGY	HMW-GS	49	CAI72574.1
P6	TASLQQPGQGQQGHYPASL	HMW-GS	42	CAA43361.1
P7	HVSVEHQAASL	HMW-GS	36	AHZ62762.1
P8	ASIVAGIGGQ	γ-gliadins	28	AGZ20271.1
P9	NIQVDPSGQVQW	γ-gliadins	57	AAF42989.1
P10	LQPQQPQQSFPQQQQPL	γ-gliadins	63	ACJ03470.1
P11	LQLQPFPQPQLPYPQPQPF	α-gliadins	63	AAZ94421.1
P12	FQPSQQNPQAQGF	α-gliadins	64	BAM08452.1
P13	RPQQPYPQPQPQY	α-gliadins	48	AHN85627.1
P14	QQYPQQQPSGSDVISISGL	ω5-gliadins	53	BAE20328.1
P15	GSSLTSIGGQ	ω1,2-gliadins	43	BAN29067.1
P16	FPHQSQQPF	ω1,2-gliadins	26	ADF58069.1

^1^ Individual peptide ion scores >40 are considered to indicate identity or extensive similarity (p < 0.05) and scores 15−40 were validated manually.

^2^ Accession number of the best match in the database National Center for Biotechnology Information (NCBInr) database.

HMW-GS, high-molecular-weight glutenin subunits; LMW-GS, low-molecular-weight glutenin subunits; underlined sequences are known to be CD-active

### Grain samples

Grains of four common wheat cultivars (cv.) (cv. Akteur, I.G. Pflanzenzucht, Munich, Germany; cv. Julius, KWS Lochow, Bergen, Germany; cv. Pamier, Lantmännen SW Seed, JK Bergen op Zoom, The Netherlands; cv. Tommi, Nordsaat Saatzucht, Langenstein, Germany), all harvested in 2013, were mixed in the ratio 1/1/1/1 (w/w/w/w) and shaken overhead (Turbula, Willy A. Bachofen Maschinenfabrik, Muttenz, Switzerland) for 24 h to obtain a homogeneous grain mixture. The wheat grain mixture was milled on a Quadrumat Junior mill (Brabender, Duisburg, Germany) and sieved to a particle size of 0.2 mm (wheat flour mixture).

### Analytical characterization of the wheat flour mixture

The crude protein content (nitrogen content x 5.7) of the wheat flour mixture was determined by the Dumas combustion method according to International Association for Cereal Science and Technology (ICC) Standard Method 167 [17] using a TruSpec Nitrogen Analyzer (Leco, Kirchheim, Germany). The moisture and ash contents were determined according to ICC Standards 110/1 [18] and 104/1 [19]. Extraction of the wheat flour mixture followed by quantitative determination of the Osborne fractions by reversed-phase (RP)-HPLC was carried out as reported earlier [20,21]. The gluten content was calculated as sum of gliadins and glutenins. The gluten protein types were calculated from the RP-HPLC absorbance area (210 nm) of each gluten protein type relative to the total absorbance area of the respective gliadin or glutenin fraction. All determinations were done in triplicates.

### Preparation of gluten reference proteins

Preparative isolation of reference gluten protein fractions and types as well as the characterization of the obtained proteins was performed as described in detail by Schalk et al. (2017) [22]. Reference gluten protein fractions (gliadins and glutenins) were isolated by modified Osborne fractionation followed by preparative RP-HPLC to isolate reference gluten protein types (ω5-, ω1,2-, α-, and γ-gliadins, high-molecular-weight (HMW), and low-molecular-weight (LMW) glutenin subunits (GS)).

### Digestion of gluten reference proteins and the quantitation of marker peptides in each reference protein type

First, the wheat flour mixture (200 mg) was defatted with *n*-pentane/ethanol (95/5, v/v; 2 x 2.0 mL) [23]. Each gluten protein type isolated from the wheat flour mixture (ω5-, ω1,2-, α-, and γ-gliadins, HMW-GS and LMW-GS; 5 mg), each gluten fraction (gliadins and glutenins; 5 mg) as well as the defatted wheat flour mixture (50 mg) were suspended in a TRIS-HCl-buffer (2.0 mL, 0.1 mol/L TRIS-HCl, pH 7.8, urea 120 mg/mL) and hydrolysed with α-chymotrypsin (enzyme-to-protein ratio of 1/200, w/w) for 24 h at 37°C. To stop the digestion, TFA (5 μL) was added [24]. The obtained peptide mixtures were purified by solid phase extraction (SPE) on Supelco DSC-C_18_ tubes (100 mg, Supelco, Steinheim, Germany). The C_18_ cartridges were conditioned with methanol (1 mL) and equilibrated with TFA (0.1%, v/v, 1 mL). After loading the peptide mixtures, the cartridges were washed with water containing TFA (0.1%, v/v, 5 x 1 mL) and the peptides were eluted stepwise with different concentrations of aqueous methanol (gluten protein types and fractions: 50% and 100%, v/v, 1 mL; wheat flour mixture: 20%, 40%, 60%, and 100%, v/v, 1 mL). The eluates were dried separately in a vacuum centrifuge (40°C, 6 h, 800 Pa) and analysed by untargeted LC-MS/MS.

For the quantitation of marker peptides, all reference gluten protein types of the wheat flour mixture were hydrolysed as described above. The labelled standard *P11 was added (75 μL; 100 μg/mL) prior to the digestion. The obtained unpurified peptide mixtures were analysed by targeted LC-MS/MS.

### Untargeted LC-MS/MS

For untargeted LC-MS/MS, an HCT-Ultra PTM iontrap MS (Bruker Daltonics, Bremen, Germany) with collision-induced dissociation (CID), was used. The MS was coupled with an UltiMate 3000 HPLC (Dionex, Idstein, Germany) system and peptide separation was performed on an Aeris Peptide 3.6 μm XB-C_18_ column (2.1 × 150, 10 nm × 2.1 mm; Phenomenex, Aschaffenburg, Germany). The MS contained a spherical iontrap with an electrospray ionization (ESI) interface running in the positive mode (capillary voltage, -4000 V; capillary exit voltage, -1500 V; skimmer voltage, 40 V). Nitrogen was used as drying (8.0 L/min, 325°C) and nebulizing gas (0.2 MPa). The LC conditions were set as follows: solvent A, FA (0.1%, v/v) in water, solvent B, FA (0.1%, v/v) in acetonitrile; gradient 0–5 min isocratic 0% B, 5–45 min linear 0–30% B, 45–55 min linear 30–50% B; 55–60 min linear 50–90% B, 60–62 min isocratic 90% B, 62–65 min linear 90–0% B, 65–72 min, isocratic 0% B; flow rate, 0.2 mL/min; injection volume, 15 μL; column temperature, 22°C. Peptides were scanned in the standard enhanced mode, the scan range was *m/z* 300 to 1500 with 13000 *m/z*/s (smart target value, 300000; target mass, 900 *m/z*; maximum acquisition time: 100 ms), and CID-MS/MS scan steps were performed on precursor ions using the AutoMS/MS mode (fragmentation amplitude, 1.0 V; collision gas, helium).

### Peptide identification

MS/MS data were converted into a Mascot generic file (*.mgf) and evaluated by means of the DataAnalysis 3.4 software (Bruker Daltonics) using the MS/MS ions search module of the Mascot software (Matrix Science, London, UK) based on the NCBI database (National Library of Medicine, Bethesda, MD, USA) (taxonomic category, *Viridiplantae*; peptide mass tolerance, ± 5 amu; product ion mass tolerance, ± 0.5 amu; peptide charges, 1+, 2+ and 3+; monoisotopic ions; variable modification, ammonia loss; enzyme, chymotrypsin; maximum missed cleavage sites, 2). Individual peptide ion scores > 40 were considered to indicate identity or extensive similarity (p < 0.05). All peptide identifications with peptide ion scores between 15 and 40 were manually validated according to Chen et al. [25].

### Identification of marker peptides

All identified peptides had to fulfill the following criteria to be acceptable as suitable marker peptides for gluten quantitation: sequence specificity for each protein type, number of amino acids (8–20), and no cysteine present in the amino acid sequence [26]. Only peptides, which fulfilled all criteria and had the highest peptide scores within one protein type, were defined as ideal candidates. For each protein type, two to three marker peptides were defined.

### Targeted LC-MS/MS

The quantitation of the wheat marker peptides P1-16 was performed on a triple-stage quadrupole mass spectrometer (TSQ Vantage, Thermo Fisher Scientific, Dreieich, Germany). For peptide separation, an UltiMate 3000 HPLC system (Dionex) was coupled to the mass spectrometer and an XBridge Peptide 3.5 μm BEH-C_18_ column (1.0 x 150 mm, 13 nm; Waters, Eschborn, Germany) was used. The LC conditions were set as follows: solvent A, FA (0.1%, v/v) in water, solvent B, FA (0.1%, v/v) in acetonitrile; gradient 0–5 min isocratic 5% B, 5–25 min linear 5–55% B, 25–30 min isocratic 90% B; 30–35 min linear 90–5% B, 35–45 min isocratic 5% B, flow rate, 0.1 mL/min; injection volume, 10 μL, column temperature, 22°C. The ion source was operated in the ESI positive mode (source parameters: spray voltage, 4500 V; vaporizer temperature, 50°C; sheath gas pressure, 40 arbitrary units (au); aux gas pressure, 5 au; capillary temperature, 300°C). The MS was operated in the timed multiple reaction monitoring (MRM) mode (retention time ± 3 min). Two MRM transitions for each marker peptide were monitored and used as quantifier (most abundant MRM transition) and qualifier. A declustering voltage of -10 V was set for all transitions. The transitions from the precursor ions of P1-16 and *P11 to the respective product ions (b- and y-fragments) and the optimised collision energies are shown in Table 2. All peptides were dissolved in FA (0.1%, v/v, 10 μg/mL). These 17 stock solutions were mixed in molar ratios n (*P11)/n (P1-16) (1+9, 1+4, 1+1, 4+1, 9+1) for calibration.

**Table 2:** Optimized LC-MS/MS parameters for the 16 wheat marker peptides. Multiple reaction monitoring (MRM) parameters of P1-16 and the isotopically labelled peptide standard (*P11) as well as the corresponding response factors (*RF*), each referred to *P11.

Peptide	Precursor ion	Product ions^1^	Collision energy	Retention time	Response factor
	[*m/z*] (charge state)	[*m/z*]	[V]	[min]	(*RF*)
P1	938.78 (2+)	595.83 (b5)^2^	12	16.7	1.721
		585.55 (y5)^3^	14		
P2	527.97 (2+)	314.01 (b3)^2^	10	12.9	1.646
		186.00 (b2)^3^	14		
P3	725.07 (2+)	852.44(y8)^2^	10	17.7	0.294
		429.22(y4)^3^	16		
P4	645.63 (2+)	313.92 (b3)^2^	14	16.7	2.341
		736.19 (y6)^3^	10		
P5	538.63 (2+)	238.97 (y2)^2^	10	13.1	2.221
		182.01 (y1)^3^	16		
P6	657.06 (2+)	172.96 (b2)^2^	24	15.1	2.714
		219.21 (y2)^3^	10		
P7	589.56 (2+)	237.05 (b2)^2^	18	13.6	0.981
		444.91 (b8^2+^)^3^	16		
P8	872.70 (2+)	431.19 (y5)^2^	24	15.5	1.502
		502.23 (y6)^3^	24		
P9	685.88 (2+)	315.52 (y2-NH_3_)^2^	20	17.1	3.159
		356.09 (b3)^3^	16		
P10	1011.42 (2+)	839.02 (y7)	18	15.5	1.126
		228.96 (y2)^3^	20		
P11	755.20 (3+)	262.96(y2)^2^	14	19.0	1.277
		973.64 (y8)^3^	10		
*P11	760.50 (3+)	278.96 (y2)^2^	14	19.0	-
		989.64 (y8)^3^	10		
P12	739.15 (2+)	647.39 (y6)^2^	12	15.0	0.582
		176.01 (b2)^3^	18		
P13	814.24 (2+)	407.12 (y3)^2^	20	14.1	0.517
		770.48 (b6)^3^	18		
P14	1016.85 (2+)	901.58 (b7)^2^	16	17.7	2.712
		476.32 (y4)^3^	14		
P15	906.72 (2+)	461.28 (y5)^2^	24	14.5	3.582
		562.32 (y6)^3^	24		
P16	558.72 (2+)	853.60 (b7)^2^	12	14.9	0.367
		262.96 (y2)^3^	24		

^1^ Charge state: 1+

^2^ Precursor to product ion transition was used as quantifier

^3^ Precursor to product ion transition was used as qualifier

### Matrix calibration

The defatted wheat flour mixture was spiked with commercially available potato flour (RUF Lebensmittelwerk KG, Quakenbrück, Germany) in different ratios (1+1, 1+3, 1+9, 1+19, 1+39, 1+200) to obtain different gluten contents. The defatted wheat flour mixture (500 mg) and all spiked samples (500 mg) were extracted with a buffered salt solution (2 x 2.0 mL 0.067 mol/L K_2_HPO_4_/KH_2_PO_4_-buffer, 0.4 mol/L NaCl, pH = 7.6) at 22°C to obtain albumins and globulins (ALGL), which were discarded. The residue was extracted with gluten extraction solvent (3 x 2 mL; 50% (v/v) 1-propanol, 0.1 mol/L TRIS-HCl, pH 7.5, 0.06 mol/l (w/v) dithiothreitol) at 60°C under nitrogen. After addition of the respective solvent, each flour suspension was vortexed for 2 min and stirred for 10 min (ALGL) or 30 min (gluten). The gluten suspensions were centrifuged for 20 min at 3550 *g* and 22°C, the supernatants were dried using a vacuum centrifuge (40°C, 6 h, 800 Pa), and re-suspended in TRIS-HCl-buffer. The standard *P11 was added (100 μL; 100 μg/mL) to the samples, followed by hydrolysis with α-chymotrypsin as described above and analysed by targeted LC-MS/MS.

### Limit of detection (LOD) and limit of quantitation (LOQ) of the MS method

The LOD and LOQ of the quantitation method for the wheat marker peptides P1-16 were determined using potato flour (RUF Lebensmittelwerk KG) as blank. The extraction procedure and chymotryptic hydrolysis were performed as described above. To determine the LOD and LOQ of the LC-MS/MS method, the gluten extract was spiked at 7 different concentrations (0.01–100 mg/kg) of each marker peptide and the samples were hydrolysed by α-chymotrypsin followed by targeted LC-MS/MS analysis. The LOD was calculated based on a signal-to noise-ratio (S/N) of 3, and the LOQ on an S/N of 10 according to Schalk et al. [24]. The noise was defined as interfering peak next to the analyte, which could have an influence on the detection of the marker peptide.

### Quantitation of marker peptides in wheat starch

The extraction and chymotryptic hydrolysis of wheat starches were performed as described above. After stopping the hydrolysis with TFA (5 μL), the samples were purified by centrifugation with a membrane filter (Amicon Ultra-4, PLGC Ultracel-PL membrane, cut-off 10 kDa; Merck Millipore, Darmstadt, Germany) to remove gelatinized starch. The peptide-containing eluates were dried using a vacuum centrifuge (40°C, 6 h, 800 Pa), dissolved in FA (0.1%, v/v, 750 μL) and analysed by targeted LC-MS/MS. The results were compared to those obtained by R5 ELISA and gel-permeation high-performance liquid chromatography with fluorescence detection (GP-HPLC-FLD) [11].

### Statistics

Pearson’s product moment correlations were calculated between contents of each peptide (P1-16) and the gluten content of the wheat flour mixture and the spiked samples. Correlation coefficients (r) were defined according to Thanhaeuser et al. [20] (r > 0.78, strong correlation; 0.67–0.78, medium correlation; 0.54–0.66, weak correlation; r < 0.54, no correlation). Statistically significant differences between the gluten contents analysed by LC-MS/MS, R5 ELISA and GP-HPLC-FLD were determined by one-way analysis of variance (ANOVA) with Tukey’s test as all pairwise multiple comparison procedure at a significance level of p < 0.05 using SigmaPlot 12.0 (Systat Software, San José, CA, USA). Furthermore, Pearson’s product moment correlations were determined between the gluten contents obtained by LC-MS/MS, R5 ELISA and GP-HPLC-FLD.

## Results and discussion

### Analytical characterization of the wheat flour mixture and preparation of reference proteins

To select marker peptides from wheat, a wheat flour mixture of four cultivars widely grown in Germany was used to include genetic variability between different cultivars [22,27]. The cultivars were selected based on their production yields relative to the total production of winter wheat, in the year 2012 in Germany to include the most relevant cultivars (cumulative production share for wheat: 16%) [28]. Additionally, the wheat mixture contained flours of three different German baking performance classes (E: elite, A: high, B: bread quality) and covered the most important HMW-GS (cv. Akteur: Ax1, Dx5, Bx7, By9, Dy10; cv. Julius: Ax1, Dx2, Bx6, By8, Dy12; cv. Pamier: Dx5, Bx7, By9, Dy10; cv. Tommi: Dx2, Bx7, By9, Dy12). The crude protein content of the wheat flour mixture was 11.3 ± 0.1%, the moisture content was 13.2 ± 0.2%, and the ash content was 0.49 ± 0.01%. The sum of gliadins (5.9 ± 0.1%) and glutenins (3.0 ± 0.0%) resulted in 8.9 ± 0.1% of gluten in the wheat flour mixture and agreed with earlier findings [20]. Well-defined reference proteins were obtained by isolation of gluten protein fractions and types from the wheat flour mixture followed by characterization according to Schalk et al. [22].

### Identification of wheat marker peptides

**Fig 1:**
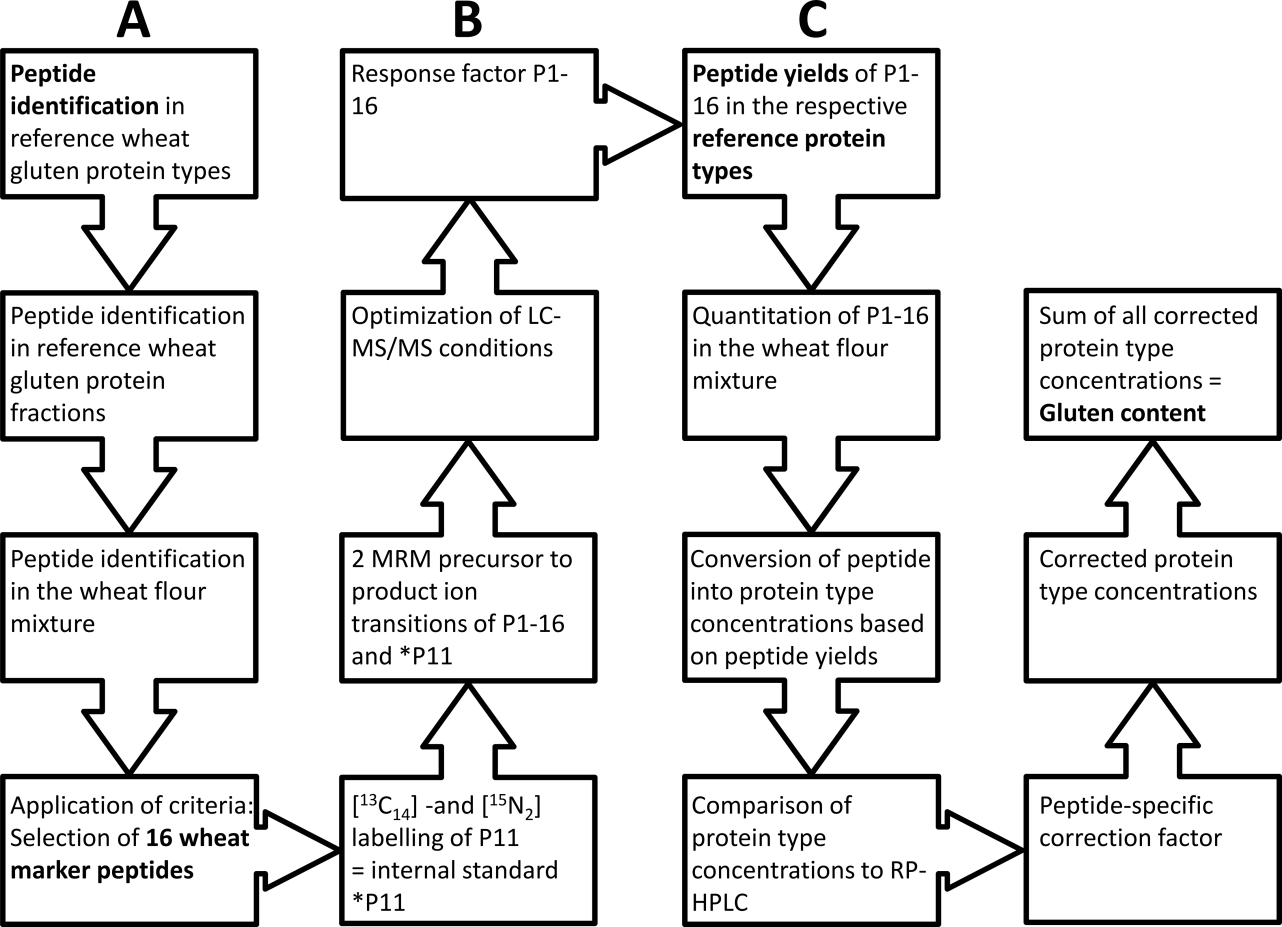
Schematic diagram showing the development of a method for the quantitation of gluten contents based on peptide yields. (A) Peptide identification and selection of 16 wheat marker peptides, (B) development of the liquid chromatography tandem mass spectrometry (LC-MS/MS) method with an isotopically labelled peptide as internal standard and optimization of the LC-MS/MS conditions, (C) quantitation of peptide yields in reference gluten protein types and conversion of peptide into protein type and gluten concentrations.

The reference gluten protein fractions (gliadins and glutenins), types (ω5-, ω1,2-, α-, and γ-gliadins, HMW-GS and LMW-GS) and the wheat flour mixture were chymotryptically hydrolysed. The obtained peptide mixtures were used to identify wheat gluten-specific peptides (wheat marker peptides) suitable for gluten quantitation (Fig 1A). The selection of suitable marker peptides was based on several criteria. The first requirement was the specificity of the peptides, i.e., that the amino acid sequences had to be characteristic for each protein type and the peptide sequences did not occur in other gluten protein types or other proteins. Secondly, the marker peptides had to consist of 8 to 20 amino acids, because shorter peptides were not specific enough and peptides longer than 20 amino acids were rather unsuitable for LC-MS/MS quantitation due to the large number of fragments and the resultant high complexity of the MS/MS spectra. Thirdly, the marker peptides should not contain cysteine residues, because of their tendency to oxidation [26]. The selection of marker peptides was not necessarily based on CD-epitope-containing peptides [29], but on peptides, which are gluten-specific and occur as widely as possible.

In the first step of identification, the isolated wheat protein types (ω5-, ω1,2-, α-, and γ-gliadins, HMW-GS and LMW-GS) were hydrolysed with α-chymotrypsin and analysed by untargeted LC-MS/MS using an iontrap MS. In total, 157 peptides were identified in all isolated wheat protein types. In each protein type the following number of peptides were identified: (ω5) 6, (ω1,2) 24, (α) 31, (γ) 11, (HMW-GS) 43, and (LMW-GS) 42. Of these, 84 peptides were potential marker peptides based on the three criteria described above. This resulted in the following number of potential marker peptides for each protein type: (ω5) 2, (ω1,2) 9, (α) 12, (γ) 10, (HMW-GS) 27, and (LMW-GS) 24. A large number of the peptides identified in ω1,2- and α-gliadins consisted of 24 to 33 amino acids and consequently did not fulfill the second criterion.

The second step of marker peptide identification was to verify this selection of 84 potential marker peptides. For this purpose, hydrolysed gliadin and glutenin fractions as well as the hydrolysed wheat flour mixture were analysed accordingly. Only peptides which were identified in hydrolysed protein types, fractions and the wheat flour mixture were suitable for gluten quantitation. 26 wheat-specific peptides were identified throughout all three stages which resulted in the following number of specific peptides for each wheat protein type: (ω5) 1, (ω1,2) 2, (α) 7, (γ) 4, (HMW-GS) 3, and (LMW-GS) 9. Based on this verified selection of peptides, two to three peptides which were detected with the highest peptide ion score in flour were defined as wheat marker peptides for each protein type. One marker peptide for each protein type was not satisfactory for gluten quantitation, because amino acids could be modified caused by deletion or exchange [30] precluding its detection by targeted LC-MS/MS. To avoid this problem, more than one marker peptide was defined to be able to detect at least one peptide for each protein type. For ω5-gliadin, only 1 marker peptide was defined, because of the low concentration in flour [21]. In total, 16 wheat marker peptides (P1-16) were defined to quantitate the amount of gluten. Table 1 shows the amino acid sequences of P1-16 with the detected peptide ion scores in flour and their specificity for each protein type. P13 was already selected for quantitation by Sealey-Voyksner et al. (2010) [12] and P11 and P13 by van den Broeck et al. (2015) [15], both of whom specifically looked for immunogenic gliadin peptides. P8, P9, P11 and P13 were also identified as candidate wheat marker peptides by Fiedler et al. (2014) [13], who also focused on the gliadin fraction. Thus, the selection of P1-16 corresponds to earlier findings in 4 out of 6 cases for α- and γ-gliadins, with the advantage that additional peptides for the other gluten protein types were added. Of those, P2, P3, P4, P7 and P13 were already identified in one sample of gluten-free wheat starch and thus, appear to be representative of gluten in different samples [11]. Further work will set about checking the validity of these wheat marker peptides across different wheat cultivars, also considering environmental variability.

### Quantitation of wheat marker peptides

**Fig 2:**
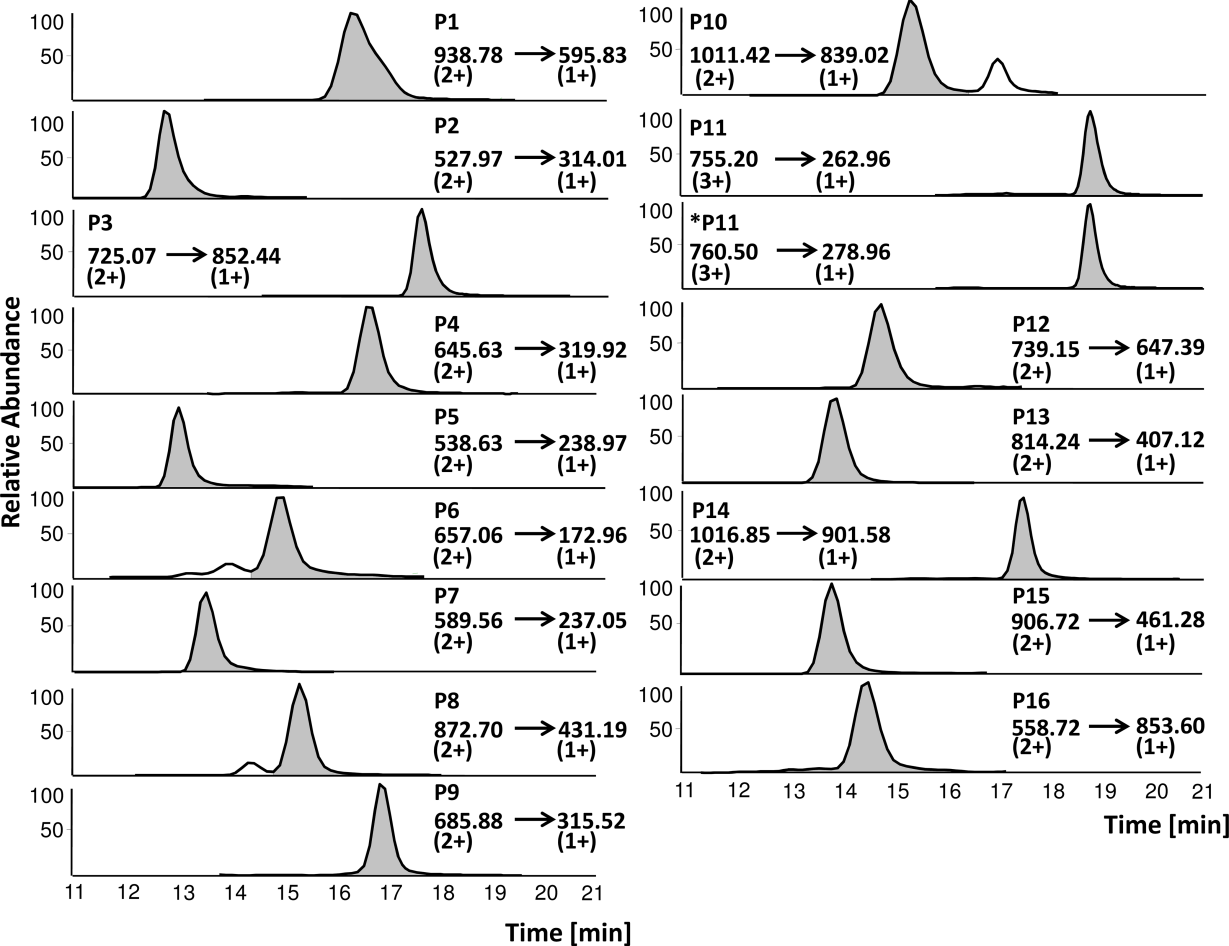
Precursor to product ion transition (*m/z*) of each marker peptide (P1-16) and the isotopically labelled standard (*P11). Marker peptides were quantitated in the respective protein type of wheat (multiple reaction monitoring mode, MRM). Two MRM transitions were monitored for each peptide and the most abundant MRM transition shown here was used for quantitation. HMW-GS, high-molecular-weight glutenin subunits; LMW-GS, low-molecular-weight glutenin subunits.

A targeted LC-MS/MS method was developed to quantitate the 16 wheat marker peptides (Fig 1B). For this purpose, P11 (LQLQPFPQPQLPYPQPQPF, monoisotopic mass 2263.2) was isotopically labelled and used as internal standard (*P11, LQLQPFPQPQLPYPQPQP*F* with F*: L-[^13^C_9_][^15^N]-phenylalanine and P*: L-[^13^C_5_][^15^N]-proline, monoisotopic mass 2279.2). P11 was chosen as internal standard, because the amino acid sequence contains the overlapping major immunogenic epitopes PFPQPQLPY (DQ2.5-glia-α1a) and PQPQLPYPQ (DQ2.5-glia-α2) [31]. P11 was isotopically labelled at the C-terminal end, because the y2-fragment (-PF) was detected as the most abundant product ion in the MS/MS spectrum and the label remained in the detected product ion in this way. All peptides except P11 were detected in the 2+ charge state as most abundant precursor ion. Only P11 and *P11 showed the highest intensity in the 3+ charge state of the precursor ion (P11, *m/z* 755.2, 3+; *P11, *m/z* 760.5, 3+). To define the most abundant transitions for MRM, the most abundant precursor ion of each P1-16 and *P11 was totally fragmented and a full MS/MS spectrum of each peptide was analysed. The most abundant MRM transition of each peptide was chosen for quantitation (quantifier) and the MRM transition following in intensity was used for qualification (qualifier) (Table 2). Fig 2 demonstrates the MRM transitions of P1-16 and *P11 which were used as quantifiers. The optimal fragmentation of each MRM transition was determined using different collision energies to induce the highest signal intensity [32] (Table 2). To confirm the identity of each marker peptide, the ratios of both monitored MRM transitions (i.e. precursor ion → quantifier to precursor ion → qualifier) were calculated in the response samples of each peptide. The stability of the determined ratios was monitored in each run and confirmed the identity of all peptides. The ratios were determined as follows: P1, 0.9; P2, 0.6; P3, 0.6; P4, 1.4; P5, 1.3; P6, 1.3; P7, 1.0; P8, 1.0; P9, 0.9; P10, 1.2; P11, 3.0, P12, 0.3, P12, 0.3; P13, 4.5; P14, 0.2; P15, 0.4, P16, 0.6, *P11, 3.0. The given ratios were constant in all analysed samples in this study.

### Calibration and quantitation

The response factor (*RF*) of each peptide was determined using the peak area ratio A (*P11)/A (P1-16) at different values of n (*P11)/n (P1-16) between 0.05 and 12.0, that lay within the linear range. The concentration of P11 was determined by stable isotope dilution assay, because analyte and internal standard had the same amino acid sequence with the only difference that *P11 was [^13^C_14_]- and [^15^N_2_]-labelled. Therefore, P11 and *P11 had the same chemical properties, retention time and ionisation behaviour and as a consequence the response factor (*RF* = 1.277) determined from the slope of the regression line was close to 1.0. P1-10 and P12-16 were also quantitated using *P11 as standard, but because they had amino acid sequences different from *P11, the response factors ranged from 0.294 to 3.582.

### Limit of detection (LOD) and limit of quantitation (LOQ)

The LOD and LOQ of the MS method to quantitate the 16 defined wheat marker peptides were determined by spiking P1-16 in seven different concentrations between 0.01 and 100 μg/g potato flour as matrix [22]. The absence of the marker peptides in hydrolysed gluten-free potato flour had been confirmed by LC-MS/MS. The LOD and LOQ for each marker peptide are shown in Table 3. The majority of peptides were detected with high sensitivity resulting in an LOD in a range between 0.2 and 3.4 μg/g and an LOQ between 0.9 and 10.5 μg/g. Only one marker peptide (P9) showed a relatively high LOD of 14.5 μg/g and three peptides (P6, 7, 9) showed a higher LOQ (16.8, 20.4 and 22.2 μg/g) as the other peptides.

**Table 3:** Limits of detection (LOD) and quantitation (LOQ) for the marker peptides P1-16 in potato flour [μg/g]. Correlation coefficients (r) were determined between peptide concentrations and gluten concentrations in the potato flour spiked to different gluten contents with the wheat flour mixture.

Peptide	Correlation coefficient (r)^1^	LOD [μg/g]	LOQ [μg/g]
P1	0.976	1.7	4.9
P2	0.912	0.2	0.9
P3	0.986	1.2	3.8
P4	0.994	0.5	5.7
P5	n.d	1.1	6.3
P6	0.943	7.5	22.2
P7	0.994	3.4	16.8
P8	0.997	0.8	3.0
P9	0.987	14.5	20.4
P10	0.985	0.8	3.0
P11	0.991	0.7	2.6
P12	0.847	3.1	10.5
P13	0.995	0.8	2.3
P14	0.970	1.9	5.6
P15	0.973	1.3	2.7
P16	n.d.	2.6	5.3

^1^ Linear Pearson product correlation. Correlation coefficients (r): 0.0 < r ≤ 0.54, no correlation; 0.54 < r ≤ 0.67, weak correlation; 0.67 < r ≤ 0.78, medium correlation; and 0.78 < r ≤ 1.0, strong correlation [20].

n.d., not determined (only detected in two spiked samples)

### Conversion of peptide into protein type concentrations

Each specific marker peptide was quantitated in the respective wheat protein type and the obtained peptide concentrations are shown in Table 4. Out of 16 peptides, 7 contained missed cleavages that are known to occur in gluten protein sequences [12,13,15], which is why the reproducibility of the chymotryptic digest of wheat protein types was confirmed first. The obtained peptide concentrations from ω5-, ω1,2-gliadins and HMW-GS (n = 3) as well as α-, γ-gliadins and LMW-GS (n = 6) showed a coefficient of variation (CV) ranging between 0.1% and 8.5% and 13 out of 16 marker peptides showed a CV of less than 5%. It appears that chymotrypsin digestion was suitable, but a profound comparison to trypsin digestion as reported by Colgrave et al. (2017) [33] would have to be done in further studies.

**Table 4:** Concentrations of the marker peptides (P1-16) in the respective protein type [μg/g] and the wheat flour mixture [μg/g]. The concentrations of protein types in flour by LC-MS/MS [%] were calculated based on peptide concentrations in the specific protein types and compared to the contents [%] quantitated by RP-HPLC. The contents determined by RP-HPLC were taken as 100% to evaluate the recovery of LC-MS/MS. Protein type concentrations had to be multiplied by the individual correction factor to adjust to recoveries of 100%.

Peptide	Protein type	Content of protein type in flour by RP-HPLC	Peptide concentration in the specific protein type	Peptide concentration in the wheat flour mixture	Content of protein type in flour by LC-MS/MS	Recovery of LC-MS/MS compared to RP-HPLC^4^	Correction factor
		[%]^1^	[μg/g]^2^	[μg/g]^1^	[%]^3^	[%]	
P1	LMW-GS	1.99 ± 0.02	10823.2 ± 162.9	29.4 ± 0.2	0.27 ± 0.03	12.0	8.29
P2	LMW-GS		11909.8 ± 310.5	24.1 ± 0.4	0.20 ± 0.01	9.6	10.47
P3	LMW-GS		4903.4 ± 38.4	21.3 ± 0.7	0.43 ± 0.02	20.5	4.85
P4	LMW-GS		8893.1 ± 411.5	224.6 ± 16.7	2.53 ± 0.18	119.2	0.84
P5	HMW-GS	0.83 ± 0.02	5251.5 ± 366.0	90.6 ± 1.2	1.73 ± 0.08	195.2	0.51
P6	HMW-GS		3286.1 ± 111.6	n.d.	n.d.	-	-
P7	HMW-GS		7542.4 ± 250.0	86.3 ± 7.9	1.14 ± 0.04	129.5	0.77
P8	γ-gliadins	1.85 ± 0.15	18703.3 ± 304.0	639.4 ± 26.11	3.42 ± 0.09	172.3	0.58
P9	γ-gliadins		16830.2 ± 716.2	477.3 ± 33.6	2.84 ± 0.39	143.9	0.69
P10	γ-gliadins		1993.4 ± 187.2	16.1 ± 1.7	0.81 ± 0.08	41.1	2.43
P11	α-gliadins	2.91 ± 0.30	5879.6 ± 57.2	137.2 ± 13.7	2.33 ± 0.22	75.3	1.33
P12	α-gliadins		3890.9 ± 104.9	18.5 ± 0.7	0.48 ± 0.03	15.3	6.47
P13	α-gliadins		9501.9 ± 219.5	8.7 ± 0.2	0.09 ± 0.01	3.0	32.33
P14	ω5-gliadins	0.51 ± 0.02	11317.8 ± 49.4	25.6 ± 2.4	0.23 ± 0.02	39.9	2.55
P15	ω1,2-gliadins	0.67 ± 0.09	5391.7 ± 467.8	86.2 ± 2.9	1.60 ± 0.12	224.1	0.45
P16	ω1,2-gliadins		793.7 ± 17.4	n.d.	n.d.	-	-

^1^ mean value ± standard deviation (n = 3)

^2^ mean value ± standard deviation (HMW-GS, ω5-, ω1,2-gliadins n = 3; LMW-GS, α-, γ-gliadins n = 6) based on the concentration of protein type

^3^ mean value ± standard deviation (n = 3) based on peptide concentrations (P1-16) in the respective protein type

^4^ The amount of protein type, which was determined by RP-HPLC, was taken as 100% to evaluate the recovery of LC-MS/MS

LMW-GS, low-molecular-weight glutenin subunits; HMW-GS, high-molecular-weight glutenin subunits; n.d., not detected due to co-elution of other similar gluten components

The peptide concentrations in the respective reference protein types formed the basis for the conversion of peptide into protein concentrations. To achieve this, the peptide yields of the chymotryptic digest obtained from a given amount of reference protein type were determined. Then, the peptide concentrations determined in the wheat flour mixture were converted into concentrations of protein type based on the respective peptide yields per protein type. In this way, a link between the obtained peptide concentrations and the respective protein types was established for all wheat marker peptides P1-16 and the efficiency of the chymotryptic digest and recovery were included in this method of calculation. In this approach, the peptide concentrations of P1-16 in the respective protein types (ω5-, ω1,2-, α-, and γ-gliadins, HMW-GS and LMW-GS) were used as reference values for the conversion of the amount of peptides determined by targeted LC-MS/MS into concentrations of wheat protein types (Fig 1C).

As an example, the calculation of the α-gliadin content using the peptide yield of P11 in the reference protein type (calculation in three steps) is explained. After the chymotryptic digest, 5879.6 μg of peptide P11 was formed from one gram of isolated α-gliadin (Table 4) (step 1). In step 2, P11 was quantitated in the wheat flour mixture and a concentration of 137.2 μg P11/g wheat flour mixture was determined. Based on a yield of 5879.6 μg P11/g α-gliadin, the wheat flour mixture contained 21.8 mg α-gliadin/g using the concentration of 137.2 μg P11/g wheat flour mixture (step 3). Then, the amount of α-gliadin in the wheat flour mixture determined by LC-MS/MS (21.8 mg/1 g) was compared to the amount of α-gliadin, which was quantitated by RP-HPLC-UV (29.1 mg/g). The amount of protein type determined by RP-HPLC-UV was taken as 100% and, thus, the recovery of LC-MS/MS was 75.3% based on peptide P11. The amount of each peptide P1-16 was converted into the concentration of the respective protein type following the same procedure, including the corresponding recoveries (Table 4). As a consequence, to calculate the amount of protein type in a real sample by LC-MS/MS, the obtained concentration had to be multiplied by the peptide-specific correction factor. The marker peptides from γ-gliadins, LMW- and HMW-GS were derived from several identical protein isoforms and therefore individual peptide correction factors were calculated. The marker peptides from α- and ω1,2-gliadins were derived from different protein isoforms and also summed up before comparison to amounts determined by RP-HPLC (S1 Table). The sum of P11, 12 and 13 yielded an α-gliadin content of 2.73%, which resulted in a recovery of 94% and a correction factor of 1.06. In case of detection of only one marker peptide from α-gliadin, the correction factor of 1.06 would overestimate the amount of α-gliadin and that is why individual peptide correction factors for P11, 12 and P13 were calculated (Table 4). The marker peptides P6 and P16 were only detected by untargeted LC-MS/MS, but the analysis of these two peptides by targeted LC-MS/MS showed interfering peaks at the same retention time (R_t_; P6, R_t_ = 15.1 min; P16, R_t_ = 14.9 min) which made their quantitation impossible in the wheat flour mixture. Therefore, the ω1,2- gliadin content was only calculated based on P15. According to van den Broeck et al. [15], the amounts of protein types were calculated based on the average molecular weight (MW) of the respective protein type as described previously [22] (S2 Table). For example, the amount of peptide P11 [mmol] was converted into the corresponding amount of α-gliadin using the average MW of α-gliadins (32286), which resulted in 0.2% α-gliadin in flour. The presented method, which considers peptide-specific yields from reference protein types and the efficiency of enzymatic digest, resulted in 1.9% α-gliadin in flour, which corresponded more accurately with the amount determined by RP-HPLC.

### Matrix calibration

**Fig 3:**
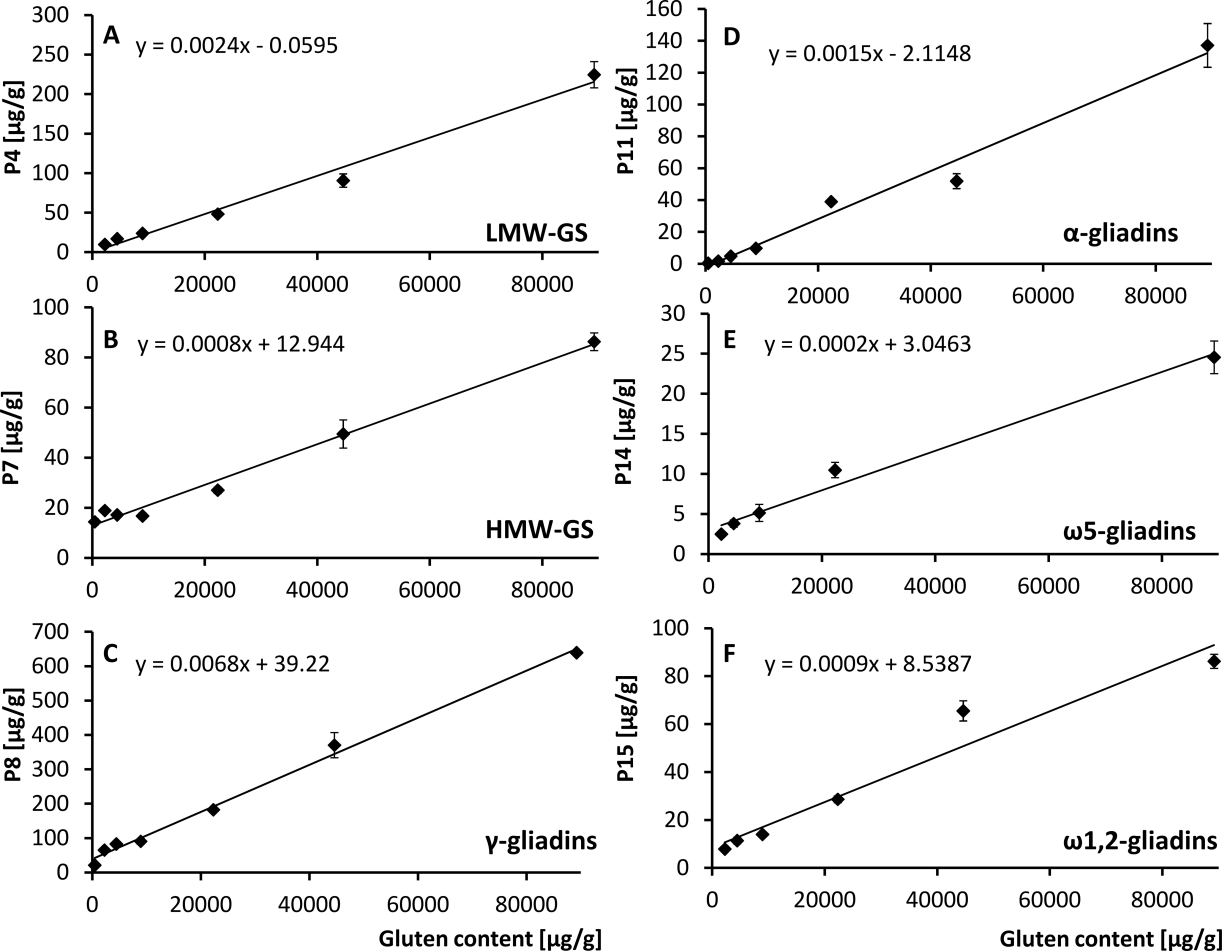
Linear Pearson correlations between gluten contents and concentrations of peptides from all wheat gluten protein types. (A) Peptide P4 from low-molecular-weight glutenin subunits (LMW-GS), (B) P7 from high-molecular-weight glutenin subunits (HMW-GS), (C) P8 from γ-gliadins, (D) P11 from α-gliadins, (E) P14 from ω5-gliadins, (F) P15 from ω1,2-gliadins. The presented peptides showed the highest correlation coefficients within the respective protein type (see Table 3).

Each marker peptide (P1-16) was determined in the wheat flour mixture with known gluten content (89200 μg gluten/g) as well as in the wheat flour mixture spiked into gluten-free potato flour to obtain different gluten contents (44600, 22300, 8920, 4460, 2230 and 446 μg gluten/g). The gluten content of the wheat flour mixture was determined by RP-HPLC as sum of gliadins and glutenins. Gluten contents of the spiked samples were calculated based on the gluten content of the wheat flour mixture and the dilution factor. A strong correlation between peptide and gluten concentrations was observed for each marker peptide with correlation coefficients (r) > 0.847 (Table 3). The marker peptides P1, 2, 4, 11, and 14 were quantitated down to a content of 2230 μg gluten/g. In the spiked sample containing 446 μg/g, these five marker peptides were below the respective LODs (Table 3). The marker peptides P3, 10, 12, and 13 were only quantitated down to 4460 μg gluten/g in the spiked sample because the peptide contents were lower than the respective LODs (Table 3) in the samples with 2230 μg gluten/g and below. The marker peptides P6, 7, 8, 9, and 15 were quantitated down to a content of 446 μg gluten/g. The lowest quantitated peptide concentration of each marker peptide lay in between the determined LOQ and LOD of each peptide, but these concentrations still lay within the linear range. Fig 3 demonstrates the correlation between the concentrations of one peptide of each wheat protein type (P4, LMW-GS; P7, HMW-GS; P8, γ-gliadins; P11, α-gliadins; P14, ω5-gliadins; P15, ω1,2-gliadins) and the gluten contents of the spiked samples which showed the highest correlation within the same protein type.

This experiment confirmed that the marker peptides were sensitively detected at low levels of μg peptide/g flour. In the wheat flour mixture, the highest peptide yield was 639.4 μg/g of P8 and all other peptides had much lower concentrations than P8 (Table 4). Due to the comparatively low peptide concentrations compared to the high gluten content (89200 μg/g) of the wheat flour mixture, it was not feasible to quantitate the marker peptides at low levels of gluten concentrations using this approach. Further work will focus on improving sample preparation and clean-up and possibly selecting other precursor to product ion transitions less prone to interference to make the method more sensitive.

### Quantitation of marker peptides in wheat starch, conversion into gluten contents and comparison to R5 ELISA and GP-HPLC-FLD

Seven wheat starches with different gluten contents were analysed by LC-MS/MS and the results compared to those obtained by sandwich R5 ELISA and GP-HPLC-FLD in a previous study [11]. Each of the methods had their own procedure to calculate the gluten content of the sample. By LC-MS/MS, the marker peptides were quantitated and selected marker peptides were used for the calculation of protein type concentrations. Afterwards, the obtained protein type concentrations were multiplied by the individual correction factor and the sum of all determined protein type concentrations resulted in the gluten content. By sandwich R5 ELISA, the gliadin content was determined and multiplied by a factor of 2 to calculate the gluten content [3]. By GP-HPLC-FLD, the concentrations of gliadins and glutenins were determined and the sum of both fractions resulted in the gluten content [11].

Only some marker peptides were detected and quantitated in all seven wheat starches (Table 5). The peptides P4 (LMW-GS), 7 (HMW-GS), 8 (γ-gliadins), 11 (α-gliadins) and 15 (ω1,2-gliadins) showed the highest correlation coefficients and the best recoveries compared to RP-HPLC within each protein type (Table 3), which is why these were selected for further calculations.

**Table 5:** Concentrations of the marker peptides [μg/g] in seven wheat starches. The wheat starches used were W4, W6, W8, W11, W13, W14 and W15 as described in Scherf et al [11]. Those marker peptides not listed had concentrations below the respective limit of detection.

*Wheat starch*			
Peptide	Protein type	Peptide concentration in wheat starch	Resulting protein type concentration
		[μg/g]^1^	[μg/g]^1^
*W4*			
P4	LMW-GS	0.7 ± 0.0	63.3 ± 2.0
P8	γ-gliadins	0.7 ± 0.0	20.1 ± 1.5
P9	γ-gliadins	120.3 ± 10.8	4669.9 ± 420.1
*W6*			
P4	LMW-GS	0.9 ± 0.1	92.2 ± 20.1
P8	γ-gliadins	0.9 ± 0.1	25.3 ± 9.7
*W8*			
P2	LMW-GS	16.9 ± 0.1	13008.2 ± 1660.1
P3	LMW-GS	7.0 ± 1.8	6544.7 ± 169.7
P4	LMW-GS	27.9 ± 2.0	2538.7 ± 169.7
P7	HMW-GS	22.9 ± 2.3	1886.7 ± 580.8
P8	γ-gliadins	107.0 ± 1.9	2874.8 ± 418.4
P11	α-gliadins	5.9 ± 0.0	1291.0 ± 85.5
P15	ω1,2-gliadins	6.7 ± 1.0	523.2 ± 78.9
*W11*			
P4	LMW-GS	3.7 ± 0.2	330.9 ± 22.0
P8	γ-gliadins	3.5 ± 0.2	102.9 ± 7.1
P9	γ-gliadins	74.1 ± 7.5	2874.6 ± 289.2
*W13*			
P9	γ-gliadins	60.0 ± 2.7	2328.4 ± 105.3
*W14*			
P4	LMW-GS	0.5 ± 0.0	43.5 ± 2.2
P9	γ-gliadins	131.8 ± 0.6	5332.7 ± 377.0
*W15*			
P4	LMW-GS	8.5 ± 0.6	755.7 ± 56.6
P7	HMW-GS	7.7 ± 1.1	743.7 ± 107.8
P8	γ-gliadins	19.0 ± 2.5	554.2 ± 71.8
P11	α-gliadins	2.3 ± 0.2	479.4 ± 40.2
P15	ω1,2-gliadins	0.7 ± 0.1	132.7 ± 10.8

^1^ mean value ± standard deviation (n = 3)

LMW-GS, low-molecular-weight glutenin subunits; HMW-GS, high-molecular-weight glutenin subunits

Peptide P4 (LMW-GS) was detected in all starches except W13 and P8 (γ-gliadins) in five out of seven starches. If the gluten content was calculated using P9, it showed significantly higher values (W13, W14) compared to the values obtained by R5 ELISA and GP-HPLC-FLD (Table 6). In W13, only peptide P9 was detected and the conversion resulted in a significantly higher gluten content compared to R5 ELISA and GP-HPLC-FLD. In contrast, the conversion of the peptide P8 concentrations into gluten contents (W4, W6, W8, W11, W15) resulted in values, which lay in the same range compared to R5 ELISA and GP-HPLC-FLD. Consequently, the concentration of peptide P9 seemed to be overestimated, which could be caused by co-elution of other similar gluten components. In wheat starch the MRM transitions of P9 showed interferences, which could explain the overestimation. Based on these results, P9 was eliminated for gluten calculation. Just two peptides (P4, 9) were detected in W14 and only P4 was used for the conversion into the gluten content, which yielded 43.7 μg gluten/g and showed a similar value compared to the other two methods. In W4 and W11, peptides P4 and P8 were used for the calculation of gluten contents, which showed similar results compared to R5 ELISA and GP-HPLC-FLD (Table 6). The gluten contents of W4, W11 and W14 were calculated based on all detected marker peptides except P9. In W8 and W15, one peptide of each protein type except ω5-gliadins was quantitated. In W15, all detected marker peptides were used for gluten calculation, because only marker peptides derived from different protein isoforms were detected. In W8, P2, P3 and P4 from LMW-GS were detected, which mainly derived from the same protein isoforms (S1 Table). The sum of all three peptides from the same protein isoforms would result in the overestimation of LMW-GS. Only P4 was used for the conversion into LMW-GS, because it gave the best recovery and correlation within this protein type and P4 was used for calculation in the other starches (W4, W6, W11, W14, W15) and therefore, provided better comparability between the different starches. The marker peptides P7, P8, P11 and P15 derived from different isoforms and were summed up. These two samples had the highest gluten contents compared to all others. The gluten content of W15 quantitated by LC-MS/MS was about 40% lower than those determined by R5 ELISA and GP-HPLC-FLD. The gluten content of W8 ranged between 9114 μg gluten/g (LC-MS/MS) and 11904 μg gluten/g (R5 ELISA) with an overall average of 10459 μg gluten/g. A significant difference was only observed between the gluten content of LC-MS/MS and R5 ELISA. This experiment showed that the lower the gluten content in wheat starch, the fewer marker peptides were quantitated, which may be caused by additional washing steps to decrease the gluten content of wheat starch [34,35]. As a result, several gluten proteins which contained the marker peptides were removed and not detected anymore in wheat starches with gluten contents below 100 μg/g. Looking at the gluten contents of all seven analysed wheat starches, the comparison of LC-MS/MS and GP-HPLC-FLD resulted in a strong correlation (r = 0.909, p < 0.005) as well as the comparison of LC-MS/MS and R5 ELISA (r = 0.919, p < 0.005). Overall, the results of the three different methods for gluten quantitation gave comparable results for W6, W8, W11 and W14. However, there was a rather large difference for W4, W13 and W15. The LC-MS/MS result for W4 lay in between those of GP-HPLC-FLD and R5 ELISA. Considering the gliadin/glutenin ratio of 0.76, it is likely that the gluten content was underestimated by R5 ELISA [11], because the gliadin content measured by ELISA is duplicated to obtain the gluten content assuming a ratio of 1. Further studies would be required to explain the difference between the two chromatographic methods, but the presence of γ-gliadins and LMW-GS as major residual gluten components in wheat starches as detected by LC-MS/MS is in line with earlier findings [11]. The very high gluten content in W13 detected by LC-MS/MS was due to the calculation based solely on P9, which was the only peptide above the LOQ, but the MRM trace showed interferences, as explained above. Therefore, the LC-MS/MS result for gluten is likely too high compared to GP-HPLC-FLD and R5 ELISA. The gluten content of W15 was lower using LC-MS/MS compared to the other two methods, although peptides from all but one gluten protein types (except ω5-gliadins) were detected. It is, however, possible, that further gluten peptides/proteins were present that had no marker peptides in their amino acid sequences. At the moment, the ELISA R5 Mendez Method is considered as the “gold standard” in gluten analysis by Codex [3], but the current state of knowledge does not provide definite answers to the question which method provides the most accurate results. Even the use of different ELISA kits resulted in significantly different gluten contents for the same wheat starch sample [36] and this issue can only be addressed by further comparative analyses and enhancement of immunological and chromatographic gluten detection methods. When considering costs and time needed for one analysis, the three methods are all quite different. The extraction procedure takes about 2 h for R5 ELISA, about 3.5 h for GP-HPLC-FLD and about 39 h for LC-MS/MS, with an additional 2 h of measurement time per sample for R5 ELISA (up to 28 samples can be run in parallel in triplicates), 0.5 h for GP-HPLC-FLD and 0.75 h for LC-MS/MS. The costs are certainly highest for LC-MS/MS, because of the expensive instrumentation and skilled personnel required to perform the experiments, but it is difficult to put a number onto the cost of one analysis. ELISA is the cheapest method in comparison, with GP-HPLC-FLD in between, but certainly closer to ELISA than to LC-MS/MS. In total, ELISA seems to be preferable to the other two methods in terms of costs and time needed.

**Table 6:** Gluten contents [μg/g] of wheat starches W4, W6, W8, W11, W13, W14 and W15. Results from different methods, LC-MS/MS, GP-HPLC-FLD and R5 ELISA, were compared.

Sample	Method		
	LC-MS/MS^1^	GP-HPLC-FLD^2^	R5 ELISA^3^
	[μg/g]	[μg/g]	[μg/g]
W4	83.4 ± 0.7^A^	158.6 ± 3.6^B^	46.8 ± 2.1^C^
W6	117.5 ± 2.8^A^	103.6 ± 2.4^B^	82.5 ± 0.5^C^
W8	9114.4 ± 901.0^A^	10371.8 ± 289.0^AB^	11903.8 ± 1560.8^B^
W11	433.8 ± 29.1^A^	442.7 ± 13.7^A^	424.4 ± 11.2^A^
W13	2328.4 ± 105.3^A^	196.0 ± 22.0^B^	88.4± 1.5^C^
W14	43.5 ± 2.2^A^	87.2 ± 3.4^B^	53.6 ± 2.1^C^
W15	2665.7 ± 206.9^A^	6543.3 ± 538.4^B^	7022.0 ± 544.4^B^

Values are given as mean ± standard deviation (n = 3)

Different capital letters designate significant differences (p < 0.05, one-way ANOVA, Tukey’s Test) between the three methods within one wheat starch sample

^1^ Gluten content expressed as sum of all determined protein type concentrations based on peptide concentrations

^2^ Gluten content expressed as sum of gliadins and glutenins [11]

^3^ Gluten content expressed as gliadins x 2 [3,11]

## Conclusion

The present study is the first to establish a link between concentrations of 16 wheat marker peptides and gluten contents using a targeted, quantitative LC-MS/MS method. This was only possible using well-characterized reference proteins for all gluten types. With this novel approach, peptide yields after chymotryptic hydrolysis were determined and enabled the conversion of peptide into protein type concentrations and, finally, gluten contents. The conversion of the concentrations of peptides P4 (LMW-GS), 7 (HMW-GS), 8 (γ-gliadins), 11 (α-gliadins) and 15 (ω1,2-gliadins) into the respective concentrations of gluten protein types resulted in recoveries of 75 to 224% compared to RP-HPLC (100%). Gluten contents expressed as sum of all determined protein types did not significantly differ to those analysed by GP-HPLC-FLD and R5 ELISA in wheat starches with high gluten contents. In samples with low amounts of gluten (< 100 μg/g), the new method showed deficiencies regarding sensitivity, which could be improved using a different MS instrument. This study also highlighted that gluten quantitation by LC-MS/MS is still not applicable in routine analyses and requires a high level of expertise to obtain accurate results. It is, however, suitable for samples where a part of gluten has been removed by processing, as shown here for wheat starches. Further work will undertake a comparison to other previously published LC-MS/MS methods for gluten quantitation, but this would require a collaborative effort of many research groups, because no single laboratory has all the different LC-MS/MS instruments available to achieve this. For this study, marker peptides for the detection of wheat gluten were identified including CD-active peptides P10 (DQ2.5-glia-γ1), P11 (DQ2.5-glia-α1a and -α2) and P13 (DQ2.5-glia-α3) [29], but the selection was not limited by this criterion, inter alia, because wheat gluten proteins are also known allergens and the presence/absence of wheat needs to be determined also in this case. More CD-active peptides will be added to the LC-MS/MS method developed here and high-throughput techniques capable of monitoring the whole set of known CD-active peptides would be ideal to comprehensively monitor the gluten-free status of foods for CD patients.

## Supporting information

S1 Table Database search on the number of protein isoforms for each marker peptide. Number of isoforms for each marker peptide (P1-16) in *Triticum aestivum* and the number of similar isoforms for each marker peptide.
(PDF)

S2 Table Concentrations of the marker peptides (P1-16) in the wheat flour mixture [μg/g and mmol]. Amounts of the respective protein types in the wheat flour mixture were calculated based on the molecular weight (MW) of the respective protein types.
(PDF)
