# Supplementary material for: Targeted liquid chromatography tandem mass spectrometry to quantitate wheat gluten using well-defined reference proteins
Source: PLoS One. 2018 Feb 9;13(2):e0192804. doi: 10.1371/journal.pone.0192804 (PMC5806900; doi:10.1371/journal.pone.0192804)
Supplement: S1 Table — Number of isoforms for each marker peptide (P1-16) in Triticum aestivum and the number of similar isoforms for each marker peptide. (PDF) [file pone.0192804.s001.pdf]

S1 Table. Database search on the number of protein isoforms for each marker peptide. Number of isoforms for each marker peptide (P1-16) in *Triticum aestivum* and the number of similar isoforms for each marker peptide.

Pep-tide	Amino acid sequence	NCBI Accession considering *Triticum aestivum*	Number of isoforms	Number of similar isoforms
P1	QQQPLPPQQTFPQQPL	AFX69668.1 / AGU91694.1 / AFL55405.1 / AGU91666.1 / AGK83314.1 / AFL55409.1 / AFG73618.1 / AAB48476.1 / ALN96394.1 / ALN96393.1 / ACX46518.1 / ACM77758.1 / ACM77757.1 / ACM77756.1 / AGU91676.1 / AGU91678.1 / AGO31303.1 / AGO31302.1 / AGO17747.1 / AGO17741.1 / AFB35204.1 / AFB35203.1 / AEI00688.1 / AEI00687.1 / AEI00686.1 / AEI00685.1 / AEI00684.1 / AED99854.1 / ABM73528.1 / ABM73529.1 / ALN96395.1 / ALN96392.1 / AGO17744.1 / AEB54986.1 / ABG76009.1 / AGU91656.1 / AFB35205.1 / ARJ58935.1 / ACC60298.1 / CAD58619.1 / ACI04536.1 / ACF93464.1 / AAP87371.1 / AGU91705. / AFB35207.1 / AFB35206.1 / ABY41262.1 / AAV92054.1 / AAV92021.1 / AAV92024.1 / AAV92053.1 / AAV92042.1 / AAV92023.1 / AAV92019.1 / AAV92027.1 / AAV92052.1 / AAV92022.1 / AAV92058.1 / AAV92066.1 / AAV92041.1 / AAV92033.1 / AAV92034.1 / AAV92044.1 / AAV92016.1 / AAV92036.1 / AAV92020.1 / AAV92047.1 / AAV92055.1 / AAV92062.1 / AAV92069.1 / AAV92039.1 / AAV92040.1 / AAS66084.1 / AAV92017.1 / AGO17751.1 / ABI21862.1 / AGU91664.1 / ABG45899.1 / ABG45900.1 / AAV92013.1 / AGU91690.1 / BAA22613.2 / AAV92012.1 / AGU91692.1 / BAA22614.2 / BAN29069.1 / AEB54987.1 / AGU91687.1 / AGU91681.1 / AGU91680.1 / AGU91679.1 / AFB35208.1 / ACF93468.1 / ACT98429.1 / AGU91708.1 / AQZ22280.1 / ACY08809.1 / ACT98428.1 / BAB78754.1 / AFG73615.1 / AQZ22279.1 / AQZ22278.1 / ABO43944.1 / BAB78755.1 / ACT98431.1 / ANU06100.1 / AGK83313.1 / AGK83163.1 / AGK83142.1 / CAD58622.1 / AGK83396.1 / AGK83307.1 / AGK83136.1 / ACR10430 / BAB78757.1 / AGK83402.1 / AFI81537.1 / AGK83309.1 / AGK83411.1 / AGK83412.1 / AGK83414.1	119	84 (P1 and P3)<br>26 (P1 and P2)<br>7 (P1 and P4)
P2	GQQPQQQQL	ACA63870.1 / ACA63868.1 / ACA63866.1 / ACA63865.1 / ACA63864.1 / ACA63863.1 / ALN96381.1 / ACZ59818.1 / ACA63867.1 / BAD12055.1 / ACY08813.1 / ACZ59819.1 / BAB78763.1 / ACT98430.1 / ACY08810.1 / ABG23190.1 / AGM38903.1 / AGE13923.1 / AGE13922.1 / ACT98425.1 / BAB78764.1 / BAB78739.1 / AQZ22273.1 / AAV92076.1 / AAV92078.1 / AAV92075.1 / AAV92080.1 /	224	26 (P2 and P1)<br>24 (P2 and P3)

AAS66083.1 / AAS10193.1 / AAV92077.1 /
AAV92079.1 / ABY58125.1 / ACK44494.1 /
ACK44494.1 / ACT98427.1 / AAS10191.1 /
AGO17755.1 / AGO17737.1 / AGO17734.1 /
AGO17730.1 / AGO17739.1 / ACP27637.1 /
AAS10188.1 / ACT98422.1 / ACY08822.1 /
CAA76890.1 / AQZ22275.1 / AQZ22274.1 /
AIR77171.1 / AGO17742.1 / AGK83318.1 /
ALN96380.1 / ALN96379.1 / AGO17752.1 /
AGO17749.1 / AGO17736.1 / AGO17753.1 /
AGO17733.1 / AEI00698.1 / AAS10189.1
ACT98423.1 / AFI81552.1 / AGO17735.1 /
AGU91673.1 / AHN55165.1 / ACT98421.1 /
AFI81533.1 / AAS10187.1 / ACP27639.1 /
AGK83329.1 / AGK83250.1 / AGK83149.1 /
AGK83172.1 / ABF93404.1 / AGK83320.1 /
AGK83343.1 / AGK83349.1 / AGK83381.1 /
BAA23162.2 / AFI81543.1 / AGK83189.1 /
AGK83201.1 / AGK83231.1 / BAB78740.1 /
BAB78741.1 / AHN55167.1 / AGO17754.1 /
AGO17750.1 / BAB78742.1 / ACA63856.1 /
ACA63873.1 / ACA63874.1 / ACA63875.1 /
ACA63857.1 / AEH31546.1 / AFU48613.1 /
AFU48612.1 / ABF93403.1 / ABF60939.1 /
CAB41921.1 / ACA63852.1 / ACA63859.1 /
BAJ09388.1 / ACA63853.1 / ACA63860.1 /
AGK83260.1 / ACA63854.1 / ACA63861.1 /
ACA63851.1 / ACA63858.1 / ACZ51336.1 /
AGK83135.1 / AGK83158.1 / AGK83179.1 /
AGK83240.1 / AGK83308.1 / AGK83356.1 /
AGK83369.1 / AGK83391.1 / AGK83395.1 /
AGK83336.1 / AGK83297.1 / AAB48479.1 /
AFH74361.1 / ACY08811.1 / AAS10192.1 /
ACT98424.1 / AGK83310.1 / ABM73527.1 /
AAS10190.1 / AQZ22276.1 / AGK83212.1 /
ABY58133.1 / ALN96398.1 / ALN96397.1 /
|AEI00680.1 / AEI00679.1 / AEI00677.1 /
ABC84366.1 / AGK83306.1 / AFI81544.1 /
AEI00675.1 / AGK83363.1 / AEI00676.1 /
AGK83378.1 / AEI00681.1 / AGK83383.1 /
AEI00682.1 / AGK83393.1 / AFI81532.1 /
AGK83134.1 / AGK83157.1 / AGK83167.1 /
AGK83188.1 / AGK83198.1 / AFI81544.1 /
AIU94495.1 / AIU94497.1 / AIU94501.1 /
AKQ62705.1 / AKQ62706.1 / AGO17731.1 /
AGK83337.1 / AGK83337.1 / AGK83330.1 /
AGK83251.1 / AGK83219.1 / AGK83344.1 /
AGK83190.1 / AGK83210.1 / AGK83159.1 /
AGK83317.1 / AGU91691.1 / AGK83139.1 /
ACZ51339.1 / AGK83264.1 / AGK83291.1 /
AGK83301.1 / BAD42431.1 / BAE96110.1 /
ABY58126.1 / ACA63855.1 / ACA63862.1 /
ACK44491.1 / BAD12056.1 / AGK83137.1 /
AGK83138.1 / AGK83207.1 / AGK83256.1 /
AGK83376.1 / AAS66085.1 / AQZ22281.1 /
AGO17758.1 / AFI81534.1 / AEI00668.1 /
AGO17762.1 / AGK83229.1 / AGK83199.1 /
AGK83147.1 / AGK83170.1 / AGK83295.1 /

		AGK83277.1 / AGK83286.1 / AGK83247.1 / AGK83269.1 / AGK83355.1 / AMY62702.1 / AGK83270.1 / AGK83148.1 / ACF93468.1 / AGU91694.1 / AGK83314.1 / AFL55409.1 / AFG73618.1 / ALN96394.1 / ACX46518.1 / ACM77756.1 / AGU91676.1 / AGO31302.1 / AGO17747.1 / AFB35203.1 / AEI00687.1 / AED99854.1 / ABM73528.1 /		
P3	VQQQIPVVQPSIL	AMY62702.1 / AFX69668.1 / ACF93468.1 / AGU91694.1 / AFL55405.1 / AFL55404.1 / AHN55176.1 / AHN55175.1 / AGU91666.1 / AGK83314.1 / AFL55409.1 / AFL55407.1 / AFG73618.1 / AAB48476.1 / ALN96394.1 / ALN96393.1 / ACX46518.1 / ACM77758.1 / ACM77757.1 / ACM77756.1 / AGU91676.1 / AGO31303.1 / AGO17747.1 / AGO17740.1 / AFB35204.1 / AFB35203.1 / AEI00688.1 / AEI00687.1 / AEI00686.1 / AEI00685.1 / AED99854.1 / ABM73528.1 / ABM73529.1 / AAB48478.1 / ALN96395.1 / ALN96392.1 / AGO17744.1 / ABG76009.1 / ACX46512.1 / AGU91656.1 / AFB35205.1 / BAB78761.1 / ARJ58935.1 / ACC60298.1 / CAD58619.1 / ACI04536.1 / ACF93464.1 / AAP87371.1 / AGU91705.1 / AGO31304.1 / AFB35207.1 / AFB35206.1 / ABY41262.1 / AAV92054.1 / AAV92021.1 / AAV92059.1 / AAV92024.1 / AAV92053.1 / AAV92042.1 / AAV92023.1 / AAV92045.1 / AAV92019.1 / AAV92027.1 / AAV92052.1 / AAV92022.1 / AAV92049.1 / AAV92058.1 / AAV92066.1 / AAV92074.1 / AAV92041.1 / AAV92033.1 / AAV92016.1 / AAV92036.1 / AAV92020.1 / AAV92055.1 / AAV92039.1 / AAV92040.1 / AAS66084.1 / AAV92017.1 / AAV92015.1 / AGO17751.1 / ABI21862.1 / BAB78746.1 / AGU91664.1 / ABG45899.1 / ABG45900.1 / AAV92032.1 / AAV92013.1 / AAV92031.1 / AAX98174.1 / AGU91690.1 / AAV92012.1 / AGU91692.1 / BAA22614.2 / BAN29069.1 / AEB54987.1 / AGU91687.1 / AGU91681.1 / AGU91680.1 / AGU91679.1 / AAB48474.1 / AFK13671.1 / BAE45621.1 / ACR10430.1 / AGO31302.1 / AEB54986.1 / ABD72601.1 / AEI00684.1 / AGU91683.1 / AGU91684.1 / AGU91700.1	111	84 (P3 and P1)<br>24 (P3 and P2)
P4	SIILQEQQQGF	AGU91698.1 / AGU91668.1 / AGU91665.1 / AGU91659.1 / ACX46517.1 / ACX46515.1 / ACX46513.1 / AGU91669.1 / AGO17768.1 / AGO17763.1 / AGO17756.1 / AGO17746.1 / AAV91995.1 / AAV91997.1 / AFK29760.1 / ALN96391.1 / ALN96390.1 / ALN96389.1 / ALN96387.1 / BAB78751.1 / ACH92775.1 / ABG23187.1 / AHN55169.1 / AGU91675.1 / AGU91662.1 / AGO31301.1 / AGO17767.1 / AGO17766.1 / AGO17765.1 / AGO17761.1 / AGO17743.1 / AFX69667.1 / AEI00696.1 / AEI00692.1 / AEI00690.1 / AEI00689.1 / AEI00664.1 / ABC84361.1 / AAO53259.1 /	87	7 (P4 and P1)

		ACH92776.1 / ABG23188.1 / AGO17770.1 / AGO17769.1 / AGO17757.1 / AAV91992.1 / AAV91994.1 / AGU91655.1 / ACX46514.1 / AGU91696.1 / BAB78753.1 / AGU91693.1 / AGU91682.1 / ACT98429.1 / AFK13667.1 / AFB35202.1 / AGU91672.1 / AFL55408.1 / AFG73617.1 / AAB48477.1 / ALN96401.1 / AGO17759.1 / AFX69669.1 / AFX69666.1 / BAB78754.1 / AED99852.1 / ADH51279.1 / AFK29758.1 / AFG73619.1 / ANU06100.1 / ACX46519.1 / AGU91697.1 / AEM45619.1 / AEM45618.1 / AEM45617.1 / AED99853.1 / AAV91993.1 / BAB78759.1 / ABY58128.1 / AAB48475.1 / ALN96408.1 / ALN96407.1 / BAB78760.1 / ACX46516.1 / AHN55166.1 / AGO31298.1 / AGO31297.1 / AAV92011.1 / AAV92010.1 / ABG45899.1		
P5	LQPGQGQQGY	BAG12019.2 / CAI72574.1 / AOZ35398.1 / AAN78346.1 / AEO45112.2 / AFP58009.1 / BAH37041.1 / AHN66476.1 / ABX89297.1 / ABF14401.1 / AAR98780.2 / AKP55623.1 / AIE47878.1 / ADF32930.1 / SCW25214.1 / SCW25211.1 / ANO59365.1 / AOZ35396.1 / SCW25213.1 / AAZ23584.1 / CAW30791.1 / AHC72160.1 / AFC75699.1 / AEP33189.1 / AEP33187.1 / AEO45110.1 / AAS60207.1 / AAQ75548.1 / AIA63996.1 / AHE80603.1 / AGS18766.1 / ABF14402.1 / ABX89298.1 / AGG55385.1 / BAH96595.1 / BAE96560.1 / AGA82319.1 / AIE47879.1 / ABQ14770.1 / AAF23506.1 / AEN55440.1 / AHI62993.1 / ABQ14771.1 / AAY86995.1 / CAC40687.1 / AHI62991.1 / AGZ95069.1 / ABG68035.1 / AHC72164.1 / AEO45111.1 / AEV53351.1 / ANO59366.1 / ABY59653.1 / ABY59654.1 / DAA06558.1 / AEP33190.1	57	12 (P5 and P7)<br>11 (P5 and P6)
P6	TASLQQPGQGQQGHYPASL	ABQ14771.1 / CAC40687.1 / AHI62991.1 / AGZ95069.1 / AHC72164.1 / ABG68035.1 / AEO45111.1 / AEV53351.1 / SCW25215.1 / AHE80602.1 / AAO74630.1 / CAA43361.1 / AAX86830.1 / SCW25212.1 / AIA63997.1 / AHI62992.1 / AOX22957.1 / AEN55440.1 / AAY86995.1 / ABX39536.1 / CAC40685.1 / AKP95632.1 / ABX64443.1 / ARJ58938.1 / AKP95633.1 / ACO56367.1 / AAR29051.1 / ACF93467.1 / DAA06556.1 / AAR29050.1 / BAF96643.1 / AHC72161.1 / AHF55812.1 / ACD44935.1 / AOZ35397.1 / ARJ58937.1 / CAC83003.1 / ABX89296.1	38	11 (P6 and P5)
P7	HVSVEHQAASL	BAG12019.2 / CAI72574.1 / AAN78346.1 / AEO45112.2 / AFP58009.1 / BAH37041.1 / AHN66476.1 / ABX89297.1 / ABF14401.1 / AAR98780.2 / AKP55623.1 / ADH04662.1 / AIE47878.1 / ADF32930.1 / CAC40686.1 / BAH96595.1	16	12 (P7 and P5)
P8	ASIVAGIGGQ	AGO17716.1 / AGO17724.1 / ACJ03464.1 / AGZ20273.1 / AGO17729.1 / AGO17727.1 /	58	39 (P8 and P9)<br>11 (P8 and P10)

		AGO17726.1 / AGO17715.1 / AGO17714.1 / AGO17705.1 / AGO17698.1 / AGO17694.1 / AGO17690.1 / AFX69687.1 / AEA52219.1 / AEA52218.1 / AEA30015.1 / AAK84779.1 / AFC75727.1 / ACJ03463.1 / ACI04088.1 / ACI04085.1 / ACI04082.1 / AAQ63858.1 / AED99848.1 / ACI04084.1 / AAF42989.1 / ACX37113.1 / AGO17697.1 / ACX37115.1 / AAK84778.1 / ADZ76045.1 / ADZ76043.1 / ACF93462.1 / AGZ20271.1 / AGO17701.1 / AGO17696.1 / AGO17693.1 / AGO17692.1 / AGO17691.1 / AFX69684.1 / AFX69683.1 / AFX69681.1 / AFX69680.1 / AFX69679.1 / ACI04087.1 / ACI04086.1 / ACI04083.1 / AAQ63860.1 / BAA11251.1 / ACI04081.1 / CAB75404.1 / AAQ63856.1 / AFC98438.1 / AFC98437.1 / AFC98436.1 / AFC98435.1 / ACX37114.1		
P9	NIQVDPSGQVQW	ACP39922.1 / AGJ50340.1 / AAK84774.1 / AGO17724.1 / ACJ03456.1 / AGZ20273.1 / AGO17729.1 / AGO17727.1 / AGO17726.1 / AGO17715.1 / AGO17714.1 / AGO17713.1 / AGO17705.1 / AGO17698.1 / AGO17694.1 / AGO17690.1 / AFX69687.1 / AEA52219.1 / AEA52218.1 / AEA30015.1 / AAK84779.1 / AFC75727.1 / ACI04088.1 / ACI04085.1 / ACI04082.1 / AED99848.1 / ACI04084.1 / AAF42989.1 / ACF93463.1 / ACF93462.1 / AGO17701.1 / AGO17696.1 / AGO17693.1 / AGO17692.1 / AGO17691.1 / AFX69684.1 / AFX69683.1 / AFX69682.1 / AFX69681.1 / AFX69680.1 / AFX69679.1 / ACJ03472.1 / AGO17703.1 / BAA11251.1 / AFC98437.1 / AFC98436.1 / AFC98435.1 / ACX37114.1 / AAK84773.1 / AAK84772.1	50	39 (P9 and P8)<br>7 (P9 and P10)
P10	LQPQQPQQSFPQQQQPL	ACX37111.1 / ACJ03470.1 / ACJ03454.1 / ACX37112.1 / ACJ03482.1 / AGZ20271.1 / ACJ03472.1 / ABO37962.1 / ACJ03442.1 / ACJ03448.1 / ACJ03445.1 / ACJ03475.1 / ACJ03476.1 / ACX37110.1 / ACJ03484.1 / ACJ03477.1 / ACJ03471.1 / ACJ03462.1 / ACJ03455.1 / AGJ50345.1 / AGJ50346.1 / ACJ03450.1 / AGZ20272.1 / AGZ20270.1 / AGZ20269.1 / AGZ20268.1 / AGZ20264.1 / AGZ20263.1 / AGZ20262.1 / AGZ20261.1 / AGO17728.1 / AGO17725.1 / AGO17723.1 / AGO17721.1 / AGO17720.1 / AGO17712.1 / AGO17711.1 / AGO17710.1 / AGO17708.1 / AFX69691.1 / AFX69689.1 / AFX69686.1 / AAK84776.1 / AFC98437.1 / AFC98435.1 / ACJ03458.1 / ACJ03469.1 / ACJ03463.1 / ACI04080.1 / ACX37116.1 / ACW82492.1 / ACJ03461.1 / ACX37117.1 / AFX69678.1 / ACJ03453.1 / ACJ03451.1 / AGO17706.1 / AGO17704.1 / AGO17700.1 / AGO17699.1 / AGJ50342.1 / AFX69676.1 / AFX69690.1 / AAK84777.1 / AGO17703.1 / AGO17722.1 / AGO17719.1 / AGO17718.1 / AGO17707.1 /	72	11 (P8 and P10)<br>7 (P10 and P9)

		AFX69688.1 / ACJ03474.1 / ACJ03473.1 / AAK84780.1 / AAK84775.1 /		
P11	LQLQPFPQPQLPYPQPQPF	AFX69638.1 / AFQ13471.1 / AFQ13466.1 / SCW25769.1 / ABQ52126.1 / AKC91140.1 / AFX69619.1 / AFQ13465.1 / AAZ94420.1 / ACD62597.1 / ABQ52119.1 / AFX69586.1 / AFX69583.1 / AAZ94421.1 / AFX69589.1 / AFX69588.1 / AKR80575.1 / BAS02413.1 / BAS02444.1	19	0
P12	FQPSQQNPQAQGF	BAM08451.1 / BAS02405.1 / BAM08459.1 / BAM08458.1 / BAM08455.1 / BAM08450.1 / BAM08452.1 / BAM08454.1 / BAM08456.1 / BAM08453.1 / CAY54134.1 / BAM08457.1 / AHY37815.1 / SCW25747.1 / SCW25745.1 / SCW25744.1 / SCW25742.1 / SCW25749.1 / SCW25743.1 / SCW25746.1 / AFX69622.1 / SCW25741.1 / SCW25764.1 / SCW25765.1 / AKC91120.1 / AKC91119.1 / AFX69639.1 / AFX69618.1 / AFX69605.1 / ABQ52117.1 / BAS02454.1 / AHN85630.1 / AFQ13467.1 / ABD85199.1 / AGO17669.1 / ABQ52122.1 / SCW25763.1 / APU92312.1	38	0
P13	RPQQPYPQPQPQY	AKC91136.1 / BAS02442.1 / AKB95606.1 / AHN85631.1 / AHN85628.1 / AFX69625.1 / AFX69587.1 / AKC91154.1 / AKC91153.1 / AFX69580.1 / AFQ13469.1 / SCW25728.1 / SCW25724.1 / AFX69579.1 / AFQ13464.1	15	0
P14	QQYPQQQPSGSDVISISGL	ALG75822.1 / BAE20328.1 / AJG03093.1 / AJG03079.1 / AJG03080.1 / AJG03078.1 / AJG03091.1 / AJG03090.1 / AJG03094.1 / ALG75828.1 / AJG03083.1 / ALG75823.1 / AJG03082.1 / ALG75830.1 / ALG75829.1 / AJG03095.1 / ALG75827.1 / ALG75831.1	18	0
P15	GSSLTSIGGQ	AGO17774.1 / BAN29067.1 / AGO17773.1 / AGZ20254.1 / AAG17702.1 / AGZ20260.1 / AGZ20257.1/ AGO17771.1 / AGO17772.1 / AGZ20259.1 / AGZ20258.1	11	0
P16	FPHQSQQPF	ADF58072.1 / ADF58071.1 / ADF58069.1	3	0
