# Supplementary material for: Targeted liquid chromatography tandem mass spectrometry to quantitate wheat gluten using well-defined reference proteins
Source: PLoS One. 2018 Feb 9;13(2):e0192804. doi: 10.1371/journal.pone.0192804 (PMC5806900; doi:10.1371/journal.pone.0192804)
Supplement: S2 Table — Amounts of the respective protein types in the wheat flour mixture were calculated based on the molecular weight (MW) of the respective protein types. (PDF) [file pone.0192804.s002.pdf]

S2 Table. Concentrations of the marker peptides (P1-16) in the wheat flour mixture [µg/g and mmol]. Amounts of the respective protein types in the wheat flour mixture were calculated based on the molecular weight (MW) of the respective protein types.

Peptide	Peptide concentration in the wheat flour mixture [µg/g]	Peptide concentration in the wheat flour mixture [mmol]	MW of protein type	Content of protein type in the wheat flour mixture by LC-MS/MS (based on MW of protein type) [%]
P1	29.4 ± 0.2	1.57×10^{-5}	39558[1]	0.06
P2	24.1 ± 0.4	2.29×10^{-5}	39558[1]	0.10
P3	21.3 ± 0.7	1.47×10^{-5}	39558[1]	0.06
P4	224.6 ± 16.7	1.74×10^{-5}	39558[1]	0.69
P5	90.6 ± 1.2	8.43×10^{-5}	87450[1]	0.74
P6	n.d.	-	87450[1]	-
P7	86.3 ± 7.9	7.33×10^{-5}	87450[1]	0.64
P8	639.4 ± 26.11	7.33×10^{-5}	32307[1]	2.37
P9	477.3 ± 33.6	3.48×10^{-5}	32307[1]	0.13
P10	16.1 ± 1.7	7.97×10^{-5}	32307[1]	0.03
P11	137.2 ± 13.7	6.06×10^{-5}	32286[2]	0.20
P12	18.5 ± 0.7	1.25×10^{-5}	32286[2]	0.04
P13	8.7 ± 0.2	5.35×10^{-5}	32286[2]	0.02
P14	25.6 ± 2.4	1.26×10^{-5}	50927[1]	0.06
P15	86.2 ± 2.9	9.51×10^{-5}	39651[1]	0.04
P16	n.d.	-	39651[1]	-

MW, molecular weight; n.d., not detected due to co-elution of other similar gluten components

[1] Schalk *et al*. (2017) [22]

[2] van den Broeck *et al*. (2015) [15]
